# Editorial: Neonatal Procedural Pain Management

**DOI:** 10.3389/fped.2021.783290

**Published:** 2021-10-27

**Authors:** Carlo V. Bellieni, Anna M. Aloisi, Kim K. Doheny, María A. Flores-Muñoz

**Affiliations:** ^1^Department of Pediatrics, University Hospital of Siena, Siena, Italy; ^2^Department of Physiology, University of Siena, Siena, Italy; ^3^Department of Neural and Behavioral Sciences, College of Medicine, The Pennsylvania State University, Hershey, PA, United States; ^4^Department of Pediatrics, College of Medicine, The Pennsylvania State University, Hershey, PA, United States; ^5^National Autonomous University of Mexico (UNAM) Pain Clinic and Palliative Care, General Hospital of Mexico, Mexico, Mexico

**Keywords:** neonatal procedural pain, pain management, newborn, non-pharmaceutical control strategies, pain and suffering

This special issue of *Frontiers in Pediatrics* dedicated to non-pharmacological interventions against neonatal procedural pain marks an important turning point in this theme of neonatal pain. In fact, until now when presenting this topic, we came to describe the non-pharmacological methods already known or those in development. Notable improvements have been made since recognizing that the newborn had not only the right to receive interventions for pain amelioration, but also that they produce an effective response to non-pharmacological pain relief treatments. In this issue we advance the topic further: identifying that well-known non-pharmacological treatments can also be drawn from cultures other than those of European and American countries, but above all we recognize it is *imperative* that they be part of a holistic vision of cures, otherwise they are less humane and less effective.

The study by Gan et al. on non-invasive acupuncture during ocular inspection is important because it combines non-invasive approaches with traditional Chinese techniques and provides appreciable results. This work is accompanied by the review by Fitri et al., on the various traditional painkilling methods, of which he concludes that only acupuncture is fairly well-studied in the world. Yet, other methods are not to be underestimated; in fact, foot massage, Yakson touch, and aromatherapy are interesting systems that integrate human presence in the procedural intervention and deserve further investigation.

Balice-Bourgois et al. then advance to a further step: to understand that it is the human environment surrounding the newborn that is a powerful analgesic tool. In fact, they demonstrate the importance of the interactions between healthcare professionals as well as their attitudes and beliefs surrounding the infant and her/his response to suffering. In particular, they report the importance of parental participation in comforting infants during invasive maneuvers and that parents desire involvement to relieve pain and suffering. Shiff et al. similarly report the importance of non-pharmacological interventions including those within the environment surrounding the infant, to reduce pain such as dimming lights, noise reduction, and the supportive presence of parents.

Hoarau et al.'s study shows the importance of human contact in increasing oral sucrose-induced analgesia, while Allegaert's review shows the significance of paracetamol in neonatal pain-relieving practice, and its limitations in removing procedural pain. These two latest works also have the importance of emphasizing that without the presence of humans, the painkiller for procedure or surgery remains insufficient, and in some cases, completely ineffective.

Therefore, the human presence is the focus of this issue on neonatal analgesia. We know that it can be explained from a physiological point of view with the activation of the analgesic pathways descending from the brain, and with the production of endorphins due to massage and skin-to-skin contact. Further, this is seen in reaction to mother's voice. This is also explained by the activation of an intramedullary blocking mechanism of the passage of the painful signal that goes from the periphery to the brain centers for a competition with peripheral tactile signals. This was emphasized in works on the effectiveness of breastfeeding and sensory saturation (1) or skin-to-skin contact where the human presence reassures and enhances resilience in the infant (2) who is already a neurobiologically social being. The infant already feels fear and anxiety in feeling abandoned, due to the presence of an already active amygdala, the center of sensitivity and anxiety control.

Providing a compassionate human presence during procedural interventions makes them less tiring for the operator (as the infant is less oppositional), as well as making the operator less mechanical and inhumane. Today there is a tendency to make each task or procedure a cold succession of actions. However, this should not apply to medicine and nursing, where humanistic factors must be considered. That is, intentions should be purposeful and rightly balanced, to respect the infant as a patient, making the intervention personalized and therefore more effective. It also is critical to acknowledge and respect the operator who feels negatively transformed if required to execute cold repetitive actions that are empty and meaningless, and therefore making them more prone to stress and burnout.

We therefore invite you to carefully and critically read this issue of *Frontiers in Pediatrics*, precisely to detect the novelty of the human and scientific approaches that embrace and enhance each other.

## Author Contributions

CB wrote the original draft of the paper. AA, KD, and MF-M made contributions. All authors approved the final version of the paper.

## Conflict of Interest

The authors declare that the research was conducted in the absence of any commercial or financial relationships that could be construed as a potential conflict of interest.

## Publisher's Note

All claims expressed in this article are solely those of the authors and do not necessarily represent those of their affiliated organizations, or those of the publisher, the editors and the reviewers. Any product that may be evaluated in this article, or claim that may be made by its manufacturer, is not guaranteed or endorsed by the publisher.
